# Determinants of adolescents’ contraceptive uptake in Ethiopia: a systematic review of literature

**DOI:** 10.1186/s40834-022-00183-y

**Published:** 2022-09-01

**Authors:** Alemayehu Gonie Mekonnen, Daniel Bogale Odo, Dabere Nigatu, Nakachew Sewnet Amare, Michael Amera Tizazu

**Affiliations:** 1 School of Nursing and Midwifery, Asrat Woldeyes Health Science Campus, Debre Berhan University, Debre Berhan, Ethiopia; 2 College of Health Sciences, Arsi University, Asela, Ethiopia; 3 School of Public Health, College of Medicine and Health Sciences, Bahir Dar University, Bahir Dar, Ethiopia

**Keywords:** Adolescents, Contraceptives, Review, Ethiopia

## Abstract

**Introduction:**

Various studies have identified different factors that affect adolescent contraceptive uptake in different parts of Ethiopia. However, varying results were reported across primary studies and those results need to be systematically collated to inform policies. Therefore, this systematic review aimed to synthesize the findings of those primary studies to obtain more robust and representative evidence about adolescent contraceptive uptake in Ethiopia.

**Methods:**

Five databases (MEDLINE via PubMed, Google Scholar, Scopus, Science Direct and CINAHL) were searched for papers published from January 2000 up to June 2021 in English. We limited our search to start on January 2000 as the health of adolescents have been given more attention after this period and to avoid time-lapsed biases. Seven studies were included in this systematic review. We used the Newcastle-Ottawa Scale and the Mixed Methods Appraisal Tool for quality assessment of the selected studies.

**Results:**

Determinants of adolescent contraceptive utilization were focused on four levels: individual, socio-cultural, healthcare service and knowledge related factors. Individual-related factors that influence adolescents’ contraceptive uptake include; being in the age group of 10–15 years, not currently enrolled in school and being from low-income families, while socio-cultural factors include: lack of discussion with family members, arranged marriage, pressure from a partner, harmful traditional practices, discussion with peer groups and sexual partners. Healthcare service-related factors include; lack of information about contraceptives during health facility visits, lack of privacy during service provision and inconvenient service hours at health facilities, and not visiting health facilities, whereas, knowledge related factors include; having knowledge of contraceptive methods and being heard about contraceptives from media. Also, the proportion of adolescent contraceptive uptake ranged from 12 to 79%.

**Conclusions:**

In this systematic, individual, socio-cultural, health-care-related, and knowledge-related characteristics have all been identified as influencing adolescents’ contraceptive uptake in Ethiopia. Hence, integrated interventions aimed at overcoming barriers to adolescent contraceptive uptake would be beneficial to improving adolescent contraceptive utilization in Ethiopia.

## Introduction

The World Health Organization (WHO) defines adolescents as individuals aged 10–19 years of age [1]. Adolescence is a transitional period from childhood to adulthood and is characterized by significant physical and psychosocial changes [1, 2]. Adolescents are sexually active during this time of transition, and they have unique needs for sexual and reproductive health (SRH) [1, 3]. Every year an estimated 16 million adolescents give birth, of which 95% of deliveries occur in developing countries [4]. In Ethiopia, 13.6% adolescent women had a child or were currently pregnant [5].

Many adolescents have been found to engage in unsafe sex, which can lead to an unintended pregnancy, an unsafe abortion, or teenage pregnancy, which further constitutes the leading cause of preventable adolescent mortality and morbidity [6–8]. Furthermore, the health of the newborn from adolescent women is also at risk of being preterm and low-birth-weight and is prone to neonatal death [9–13]. According to the recent studies, female adolescents who engaged in unsafe sex were more likely to die as compared to female adults. This is because adolescents have immature reproductive organs or lack of access and poor utilization of contraception [2, 5]. When adolescents have restricted access to contraceptives, their wellbeing and autonomy could also be deprived [11]. Adolescents who are unable to make an informed decision about their pregnancy could have a negative consequences [5]. Since they frequently lack the financial resources to care for their newborn, they dropped attending school [9, 10]. Despite the fact that many other factors also played a role in the low uptake of contraceptive methods, the main factors that influence adolescents from using contraceptives were anticipated stigma due to social norms and negative beliefs about contraception [6], fear of being seen by others and embracement in seeking contraceptives [14].

Ethiopia has made considerable efforts on family planning programs over the past 20 years and the commodities are free of charges in all health facilities [15]. The national modern contraceptive prevalence rate increased encouragingly from 8% in 2000 to 36% in 2016 [16]. Despite these efforts, the national unmet need for modern contraceptive were reported to be 22%, which was lower than the global FP targets [16]. This is likely because adolescents, the largest segment of the population in Ethiopia, are excluded from FP intervention initiatives [17, 18]. Primary healthcare units has not been tied enough to deliver ideal contraceptive services for the majority of adolescents, as a result, their needs for contraception are frequently ignored [19–21].

A number of studies have reported different factors that affect adolescent contraceptive uptake in different parts of the country [22–28]. However, these studies are not consistent in terms of size, scope and geographic coverage. Additionally, varying results were stated across individual studies and these results need to be systematically collated so that easy to inform policies. Therefore, this systematic review aimed to synthesize the findings of these primary studies to obtain more robust and representative evidence about adolescents’ contraceptive uptake in Ethiopia.

## Main text

### Search strategy

We used the Preferred Reporting Items for Systematic Reviews and Meta-Analyses (PRISMA) [29] to answer the research questions: what are the determinants of adolescents’ contraceptive uptake in Ethiopia? Five databases (MEDLINE via PubMed, Google Scholar, Scopus, Science Direct and CINAHL) were searched for papers published from January 2000 up to June 2021 in English. We limited our search to start on January 2000 as the health of adolescents have been given more attention after this period and to avoid time-lapsed biases. The search was supplemented by hand searching. A broad search was purposefully conducted to confirm all papers would be retrieved.

The following search terms were employed to search for articles from major databases mentioned above: social *OR* cultural *OR* demographic *AND* determinants *OR* factors *OR* barriers *AND* adolescents *OR* teenage *AND* family planning *OR* contraceptive *AND* uptake *OR* utilization OR use *AND* Ethiopia. As indicated above, alternative keywords were combined using the Boolean operator ‘*OR*’ to ensure all possible variations were captured; the search has then combined with ‘*AND*’ to narrow the search. The following limits were applied: English, full text online, and published between January 2000 and June 2021. We also limited our search to peer-reviewed literature as it guarantees quality checks.

### Inclusion and exclusion criteria

Papers were included if they were primary studies of cross-sectional, case-control, cohort, mixed-method and qualitative studies; had a focus on adolescent family planning or contraception; were published in English from January 2000 to June 2021; were available in full text online, and conducted in Ethiopia. Papers were excluded if they included youths (aged 15–24 years), systematic and traditional reviews.

### Data extraction

Three reviewers (AGM, NSA and MAT) independently searched and screened the titles and abstracts against the inclusion/exclusion criteria. Articles found suitable by title and abstract were undergone for full-text review. All authors (AGM, DN, DBO, NSA and MAT) reviewed all of the full texts that were retrieved, and the data were extracted into a summary table. Disagreements at each step of screening were resolved through discussion. Authors, year of publication, study design and setting, characteristics of participant (age group, study population and sample size), the proportion of contraceptive utilization were extracted. We collected data on the proportion of contraceptive uptake by adolescents, determinant variables of adolescent contraceptives and other main findings of the analysis of the included studies (Table 1).

**Table 1:** Characteristics of the included studies June 2021

Author	Year	Study Design	Study setting	Participant Characteristics			Proportion of contra-ceptive uptake	Reported factors influencing the uptake of adolescent contraceptive
				Age group	Study population	Sample size		
Feleke SA et al. [22]	2012	Community-based cross-sectional study	Gondar town, Northwest Ethiopia	15–19	Both male and female adolescents	1290	79.5%	Educational status of adolescents, discussion with family/relatives, peer groups, sexual partners and teachers were significantly associated with FP service utilization.
Abajobir AA, Seme A [26]	2014	Community-based cross-sectional study	Machakel district, East-Gojjam	10–19	Both male and female adolescents	415	21.5	Being in the age group of 10–15 years and lack of basic knowledge of SRH. Additionally, parent disapproval and pressure from partners deterred adolescents from using FP.
Hidata F et al. [28]	2015	Institutional based cross-sectional study	Toke Kutaye Woreda, West Shoa zone		Both male and female adolescents	1076	40.3	Discussion with boyfriend or girlfriend and knowing of contraceptive methods were reported as factors of adolescent FP use.
Olika AK et al. [23]	2016	Survey	Secondary analysis from a national survey	15–19	Female adolescents	504	39.6%	Wealth status of adolescents’ families, educational status of adolescents and information about FP during their health facility visits were factors associated with contraceptive use.
Ansha MG et al. [25]	2017	Community-based cross-sectional study	Anchar District, Eastern Ethiopia	15–19	Both male and female adolescents	402	39.3%	Lack of adolescent SRH services, harmful traditional practices, lack of privacy and inconvenient service hour were reasons for not utilizing FP among adolescents. Additionally, religious opposition, lack of knowledge of how to use contraceptive methods were reported as reasons.
Ketema H, Erulkar A [24]	2018	Qualitative study	Beneshangul-gumuz region	18–24	Female adolescents	16	NA	The power dynamics within arranged marriages and partner approval were the biggest factors influencing adolescent FP use.
Abebe HT et al. [27]	2020	Community-based cross-sectional study	Tigray region	15–19	Female adolescents	1755	12.3	Being young age, educational level, attending school, being married, being informed about contraceptives through media, health facility visits, having a partner were the most important determinants for use of contraceptives

### Quality appraisal of the included studies

We used the Newcastle-Ottawa Scale (the most widely used guideline for reporting observational studies) [30] and the Mixed Methods Appraisal Tool (used for reporting qualitative and mixed methods studies) for quality assessment of the selected studies [31]. Each element of quality assessment was labelled as; 1 = a criterion was met and 0 = a criterion was not met. A study was considered a very good study when the sum of the criteria is 9–10, a good study when the sum of the criteria is 7–8, and satisfactory when the sum of the criteria is 5–6. All the included studies scored above 7 and are included in the analysis.

## Results

### Characteristics of included studies

**Fig. 1:**
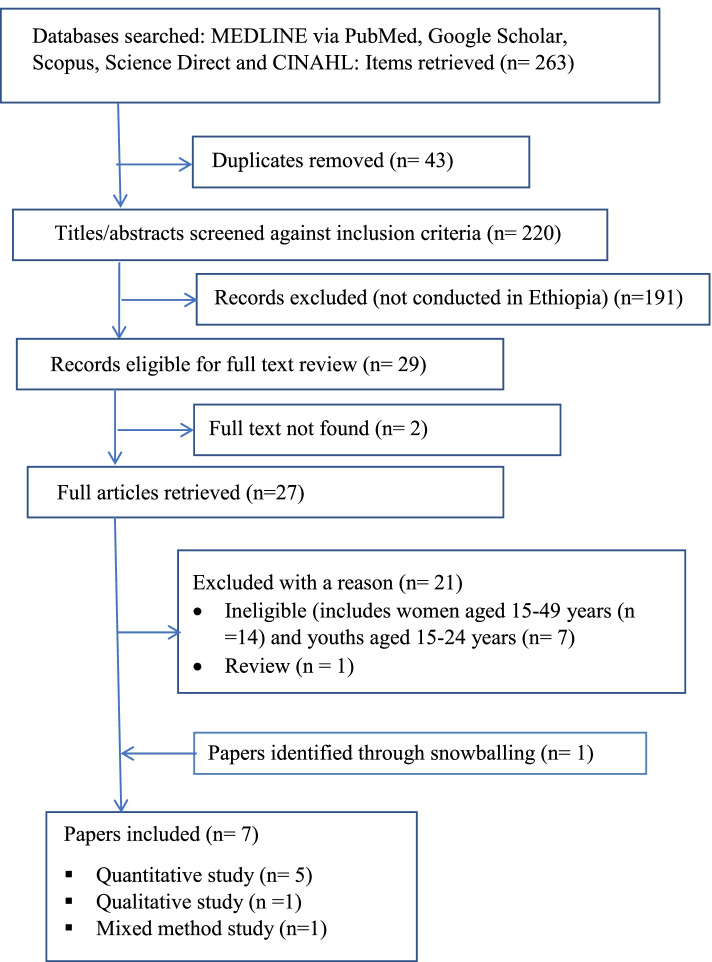
PRISMA diagram of the search process, June 2021

Figure 1 shows a flowchart of the search and results. The initial search yielded 263 records and 43 duplicates were removed. Overall, 191 papers were removed after the screening of titles and abstracts against the inclusion/exclusion criteria. Of the 29 papers remaining, 27 were retrieved in full text and assessed against the inclusion/exclusion criteria; the full texts of the two papers could not be found despite our request to the corresponding authors through email (Fig. 1).

Another 21 papers were excluded in this step, and 6 papers met the inclusion criteria. The reference lists of the included papers were scanned to identify any additional papers which may not have been captured in the initial search: 1 paper was included and making 7 eligible studies for the final analysis. Of seven included papers, five studies were quantitative [22, 23, 25, 27, 28], one study was qualitative [24] and one study was a mixed-method approach [26].

### Heterogeneity test and publication bias

The included studies were evaluated for heterogeneity and publication bias. Accordingly, the heterogeneity test showed considerable heterogeneity among studies and the true variability among the seven studies other than chance was 99.8% (I^2^ = 99.8%). The publication bias was checked by Egger’s test and the test shows no evidence of publication bias among included studies (*p* = 0.281) (Table 2). Due to the existence of considerable heterogeneity and true variability among the included studies, we decided to conduct literature review rather than meta-analysis.

**Table 2:**
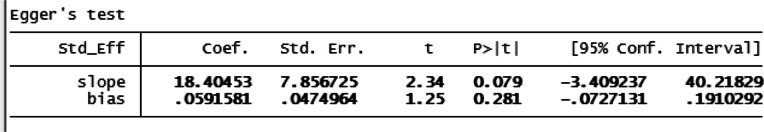
Egger’s test for small-study effects and publication bias among studies, June 2021

### Determinants of adolescents’ contraceptive uptake

**Fig. 2:**
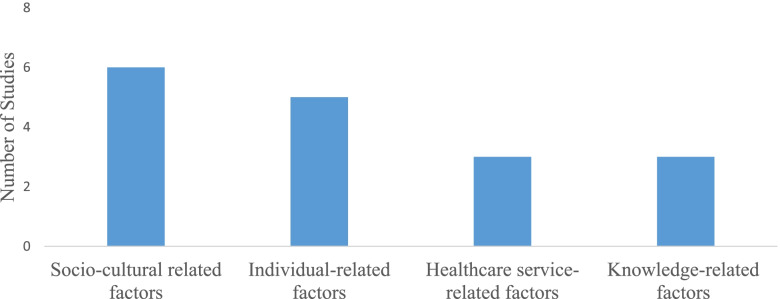
Summaries of determinants of adolescents’ contraceptive uptake in Ethiopia, June 2021

We categorized the findings of the included studies into four thematic summaries based on their similarities. These determinants are related to individual, socio-cultural, knowledge about contraceptive methods and healthcare service-related factors (Fig. 2).

### Individual-related factors

From seven studies that examined determinants of adolescent contraceptive uptake, educational status of adolescents was the main factor influencing the uptake of contraceptives as reported in three studies [22, 23, 27]. Adolescents whose age group is 10–15 years [26], those who are not currently enrolled in school [27], and those from low-income families [23] were less likely to use modern contraceptive methods.

### Socio-cultural related factors

Socio-cultural related factors are the most commonly stated determinants of adolescent contraceptive uptake and are mentioned in six studies [22–27]. Discussion with family/relatives [22], being married [27], arranged marriages and partner approval [24], parent disapproval and pressure from partners [26] having a partner [27], harmful traditional practices [25], discussion with peer groups (friends) [22, 28], and with sexual partners and teachers [22] were reported as the most important determinants for adolescent contraceptive uptake.

### Healthcare service-related factors

Healthcare service-related reasons that influence the uptake of contraceptives by adolescents were raised in three studies [23, 25, 28]. Lack of information about contraceptives during health facility visits [23], lack of access to SRH services for adolescents, lack of privacy and inconvenient service hour at health facilities [25], and not visiting health facilities [28] were negatively associated with adolescent contraceptive utilization.

### Knowledge related factors

Having knowledge of contraceptive methods [28] and basic knowledge of SRH [26], and being heard about contraceptives from media [27] were knowledge related determinants of adolescent contraceptive uptake that were mentioned in three studies, and found to be positively associated with adolescent contraceptive utilization.

### The estimates of adolescents’ contraceptive uptake

The second aim of this review was to describe the proportion of adolescent contraceptive utilization in Ethiopia, by using proportions measured in primary studies. The percentage of the variability in effect estimates (heterogeneity) between the studies was assessed using the I^2^ test. The results confirm that there is a statistical source of heterogeneity among the included studies in which the estimated points of each study are within the confidence interval of the pooled estimate. Since the included studies have a significant heterogeneity, the pooled proportion of adolescent contraceptive uptake may be less reliable, thus we simply describe the proportion of each study with its range. As a result, the proportion of adolescent contraceptive uptake varied in different parts of the country: 79% in Gondar twon (95% CI = 77,82)) [22], 21% in Machakel district (95% CI = 18, 26) [26], 40% in Toke Kutaye district (95% CI = 37,43) [28], 39% in Anchar district (95% CI = 35,44) [25], and 12% in Tigray region (95% CI = 11,14) [27].

## Discussions

We systematically reviewed the determinants of adolescent contraceptive uptake and described the proportion of contraceptive utilization in Ethiopia where adolescent contraceptive use remains very low [32]. Summarizing determinants of adolescents’ contraceptive utilization is critical for improving the well-being of the adolescent population, a population segment that is often underrepresented in most studies of modern contraception [27, 28]. The results in this review are based on seven primary studies that had been published within the last twenty years. Those included studies examined determinants of adolescent contraceptive uptake and reported its proportion in different parts of the country. We found that individual, socio-cultural, healthcare service and knowledge related factors were highlighted in the included studies as factors determining adolescent contraceptive uptake in Ethiopia.

In this review, the proportion of adolescent contraceptive uptake ranged from 12% [27] to 79% [22]. In line with our review, a mixed-effects multilevel analysis of data from 29 demographic and health surveys conducted in sub-Saharan Africa reported that, on average, 24.7% of adolescents utilized modern contraceptive methods [33]. Zhihui Li et al. in their review also reported that 31.6% of adolescent girls utilized modern contraceptives [34]. It can therefore be assumed that the proportion of adolescent contraceptive uptake still remains very low and needs integrated work to avoid unwanted adolescent pregnancies and the associated complications. This could be beneficial not only for adolescents but also their newborns and societies as a whole [32].

In this review, individual factors such as educational status of adolescents [22, 23, 27], being in the age group of 10–15 years [26], not currently enrolled in school [27] and being from low-income families [23] were less likely to use modern contraceptive methods. On the other hand, completing high school education and belonging to the highest wealth quintile families have more access to modern contraceptive information that can promote contraceptive use [13, 35]. Evidence also suggested that having a good education and an improved income status of adolescents can contribute to the improvement of contraceptive uptake by reducing gender inequality and promoting discussion with their partners or relatives, which in turn increase their utilization of contraceptive methods [12, 36]. It is also important to note that when adolescents are educated and have financial support from their families, they are less likely to be influenced by peer groups and have the opportunity to decide their fertility independently [23]. On the other hand, being lower grade and young age can demote the contraceptive use as those adolescents have limited sources of information and access to contraceptive services.

According to this review, discussion with family members [22], being married or having a partner [27], discussion with peers (friends) [22, 28], sexual partners and teachers [22] were positively associated with adolescents’ contraceptive uptake. On the other hand, arranged marriages and partner approval [24], parent disapproval and pressure from partners [26] and harmful traditional practices [25] were found to deter adolescents from using contraceptive methods. Like in Ethiopia [24, 26], the influence of social norms about sexual activity and negative beliefs about contraceptive methods and feel embracement at seeking contraceptive methods were reported as the major determinants of adolescent contraceptive uptake in Mali [37], Kenya [38], Ghana [10] and in other low-and middle-income countries [39]. Barriers to adolescent contraceptive uptake are not only restricted at the community level, they also exist among healthcare providers. For example, disapproving attitudes such as judgmental and unfriendly service provision, which could have a negative impact on contraceptive service use by adolescents, were reported in previous studies in Nigeria and Tanzania [13, 32].

Healthcare service-related reasons that influence adolescent contraceptive uptake were raised in three studies [23, 25, 28]. These include lack of information about contraceptive methods during health facility visits [23], lack of access to SRH services for adolescents, lack of privacy and inconvenient service hour at health facilities [25], and not visiting health facilities [28]. The finding of this review was similar to those reported in Ghana [9], Uganda [3] and Mali [37]. This indicates that improving the quality of family planning services offered to adolescents and creating a conducive environment at health facilities could play an important role in the initiation and the continuation of contraceptive method use [40].

Knowing contraceptive methods [28] and having basic knowledge of SRH [26], and being informed about contraceptives through media [27] were knowledge related factors that were mentioned in three studies and were found to be positively impacted adolescent contraceptive use. The role of knowing about youth-friendly services in our review was corroborated by these earlier findings [5]. This could signal how adolescent’s knowledge of contraceptive methods can cause positive behavioural change and can increase their demand for contraceptive use. Consequently, it is important to make adolescents more informed of contraceptive methods and the additional health risks that they face during pregnancy. Despite its strength, this systematic review may have some limitations. This study is based only on published studies and important data might be missed from unpublished studies.

## Conclusions

In this systematic, individual, socio-cultural, health-care-related, and knowledge-related characteristics have all been identified as influencing adolescent contraceptive uptake in Ethiopia. Hence, integrated interventions aimed at overcoming barriers to adolescent contraceptive uptake would be beneficial to improving adolescent contraceptive utilization in Ethiopia.

## Data Availability

All data generated/analyzed during this study are included in this published article. Besides, the row datasets will be available from the corresponding author on a reasonable request.

## Abbreviations

AOR: Adjusted Odds Ratio
CI: Confidence Interval
FP: Family Planning
NA: Not applicable
SRH: Sexual and Reproductive Health
PRISMA: Preferred Reporting Items for Systematic Reviews and Meta-Analyses
WHO: World Health Organization

## References

[1] WHO (2018). WHO recommendations on adolescent sexual and reproductive health and rights.

[2] Mulugeta B, Girma M, Kejela G, Meskel FG, Andarge E, Zerihun E (2019). Assessment of youth-friendly service quality and associated factors at public health facilities in southern Ethiopia: a facility-based cross-sectional study. Biomed Res Int.

[3] Sserwanja Q, Musaba MW, Mukunya D (2021). Prevalence and factors associated with modern contraceptives utilization among female adolescents in Uganda. BMC Womens Health.

[4] Nove A, Matthews Z, Neal S, Camacho AV (2014). Maternal mortality in adolescents compared with women of other ages: evidence from 144 countries. Lancet Glob Health.

[5] Alemayehu T, Haider J, Habte D. Determinants of adolescent fertility in Ethiopia. Ethiop J Health Dev. 2010;24(1) https://www.ajol.info/index.php/ejhd/article/view/62942.

[6] Jain A, Ismail H, Tobey E, Erulkar A (2019). Stigma as a barrier to family planning use among married youth in Ethiopia. J Biosoc Sci.

[7] Chandra-Mouli (2017). A never-before opportunity to strengthen investment and action on adolescent contraception, and what we must do to make full use of it. Reprod Health.

[8] Motuma A, Syre T, Egata G, Kenay A (2016). Utilization of youth-friendly services and associated factors among youth in Harar town, East Ethiopia: a mixed-method study. BMC Health Serv Res.

[9] Yakubu I, Garmaroudi G, Sadeghi R, Tol A, Yekaninejad MS, Yidana A (2019). Assessing the impact of an educational intervention program on sexual abstinence based on the health belief model amongst adolescent girls in northern Ghana, a cluster randomised control trial. Reprod Health.

[10] Oppong FB, Logo DD, Agbedra SY, Adomah AA, Amenyaglo S, Arhin-Wiredu K, Afari-Asiedu S, Ayuurebobi K (2021). Determinants of contraceptive use among sexually active unmarried adolescent girls and young women aged 15-24 years in Ghana: a nationally representative cross-sectional study. BMJ Open.

[11] Muntean N, Kereta W, Mitchell KR (2015). Addressing the sexual and reproductive health needs of young people in Ethiopia: an analysis of the current situation. Afr J Reprod Health.

[12] Gahungu J, Vahdaninia M, Regmi PR (2021). The unmet needs for modern family planning methods among postpartum women in sub-Saharan Africa: a systematic review of the literature. Reprod Health.

[13] Nsanya MK, Atchison CJ, Bottomley C, Doyle AM, Kapiga SH (2019). Modern contraceptive use among sexually active women aged 15–19 years in North-Western Tanzania: results from the adolescent 360 (A360) baseline survey. BMJ Open.

[14] Munea AM, Alene GD, Debelew GT (2020). Quality of youth-friendly sexual and reproductive health services in west Gojjam zone, north West Ethiopia: with special reference to the application of the Donabedian model. BMC Health Serv Res.

[15] Gonie A, Wudneh A, Nigatu D, Dendir Z (2018). Determinants of family planning use among married women in bale eco-region, Southeast Ethiopia: a community-based study. BMC Womens Health.

[16] Central statistical agency (CSA) [Ethiopia] and ICF (2016). Ethiopia demographic and health survey 2016.

[17] Tigabu S, Demelew T, Seid A, Sime B, Manyazewal T (2018). Socioeconomic and religious differentials in contraceptive uptake in western Ethiopia: a mixed-methods phenomenological study. BMC Womens Health.

[18] Binu W, Marama T, Gerbaba M, Sinaga M (2018). Sexual and reproductive health services utilization and associated factors among secondary school students in Nekemte town, Ethiopia. Reprod Health.

[19] Dingeta T, Oljira L, Worku A, Berhane Y (2021). Low contraceptive utilization among young married women is associated with perceived social norms and belief in contraceptive myths in rural Ethiopia. PLoS One.

[20] Kettema WG, Aynalem GL, Yismaw AE, Degu AW (2020). Modern contraceptive utilization and determinant factors among street reproductive-aged women in Amhara regional state zonal towns, Northwest Ethiopia: a community-based study. Int J Reprod Med.

[21] Abate AT, Ayisa AA (2019). Reproductive health services utilization and its associated factors among secondary school youths in Woreta town, South Gondar, Northwest Ethiopia: a cross-sectional study. BMC Res Notes.

[22] Feleke SA, Koye DN, Demssie AF, Mengesha ZB (2013). Reproductive health service utilization and associated factors among adolescents (15–19 years old) in Gondar town, Northwest Ethiopia. BMC Health Serv Res.

[23] Olika AK, Kitila SB, Terfa YB, Olika AK (2021). Contraceptive use among sexually active female adolescents in Ethiopia: trends and determinants from national demographic and health surveys. Reprod Health.

[24] Ketema H, Erulkar A (2018). Married adolescents and family planning in rural Ethiopia: understanding barriers and opportunities. Afr J Reprod Health.

[25] Ansha MG, Bosho CJ, Jaleta FT (2017). Reproductive health services utilization and associated factors among adolescents in Anchar District, East Ethiopia. J Family Reprod Health.

[26] Abajobir AA, Seme A (2014). Reproductive health knowledge and services utilization among rural adolescents in east Gojjam zone, Ethiopia: a community-based cross-sectional study. BMC Health Serv Res.

[27] Abebe HT, Belachew AB, Gebretsadik LG, Berhe YZ, Gebru HB, Kahsay AB, et al. Contraceptive use and its determinants among adolescent women in Tigray, Ethiopia: multilevel modeling. Int J Adolesc Med Health. 2020. 10.1515/ijamh-2020-0107/html. 10.1515/ijamh-2020-0107 32881709

[28] Hidata F, Worku A, Urgessa F (2015). Contraception use and factors contributing to non-use of contraception among in-school adolescents in toke Kutaye Woreda, west Shoa zone, Oromia regional state, Ethiopia. J Pregnancy Child Health.

[29] Moher D, Liberati A, Tetzlaff J, Altman DG, Prisma Group (2009). Preferred reporting items for systematic reviews and meta-analyses: the PRISMA statement. PLoS Med.

[30] Luchini C, Stubbs B, Solmi M, Veronese N (2017). Assessing the quality of studies in meta-analyses: advantages and limitations of the Newcastle Ottawa scale. World J Meta Anal.

[31] Pace R, Pluye P, Bartlett G, Macaulay AC, Salsberg J, Jagosh J, Seller R (2012). Testing the reliability and efficiency of the pilot. Mixed-methods appraisal tool (MMAT) for systematic mixed studies review. Int J Nurs Stud.

[32] Atchison CJ, Mulhern E, Kapiga S, Nsanya MK, Crawford EE, Mussa M, Bottomley C, Hargreaves JR, Doyle AM (2018). Evaluating the impact of an intervention to increase uptake of modern contraceptives among adolescent girls (15–19 years) in Nigeria, Ethiopia and Tanzania: the adolescents 360 quasi-experimental study protocol. BMJ Open.

[33] Ahinkorah BO (2020). Predictors of modern contraceptive use among adolescent girls and young women in sub-Saharan Africa: a mixed-effects multilevel analysis of data from 29 demographic and health surveys. Contracept Reprod Med.

[34] Li Z, Patton G, Sabet F, Zhou Z, Subramanian SV, Lu C (2020). Contraceptive use in adolescent girls and adult women in low-and middle-income countries. JAMA Netw Open.

[35] Hounton S, Barros AJ, Amouzou A, Shiferaw S, Maïga A, Akinyemi A, Friedman H, Koroma D (2015). Patterns and trends of contraceptive use among sexually active adolescents in Burkina Faso, Ethiopia, and Nigeria: evidence from cross-sectional studies. Glob Health Action.

[36] Banke-Thomas OE, Banke-Thomas AO, Ameh CA (2017). Factors influencing utilisation of maternal health services by adolescent mothers in low-and middle-income countries: a systematic review. BMC Pregnancy Childbirth.

[37] Ahinkorah BO (2002). Individual and community-level factors associated with modern contraceptive use among adolescent girls and young women in Mali: a mixed-effects multilevel analysis of the 2018 Mali demographic and health survey. Contracept Reprod Med.

[38] Kumar M, Huang KY, Othieno C, Wamalwa D, Madeghe B, Osok J, Kahonge SN, Nato J, McKay MM (2018). Adolescent pregnancy and challenges in Kenyan context: perspectives from multiple community stakeholders. Global Soc Welfare.

[39] Coll CD, Ewerling F, Hellwig F, De Barros AJ (2019). Contraception in adolescence: the influence of parity and marital status on contraceptive use in 73 low-and middle-income countries. Reprod Health.

[40] Blanc AK, Tsui AO, Croft TN, Trevitt JL (2009). Patterns and trends in adolescents' contraceptive use and discontinuation in developing countries and comparisons with adult women. Int Perspect Sex Reprod Health.

